# An Interesting Case of a 57-Year-Old Male with an Upper Gastrointestinal Bleeding and Obstructive Uropathy with Bilateral Hydronephrosis Diagnosed with Systemic Mucormycosis

**DOI:** 10.1155/2018/6283701

**Published:** 2018-06-20

**Authors:** Dora E. Izaguirre-Anariba, Felicia Chee, Zeyar Thet, Jesus Lanza

**Affiliations:** Wyckoff Heights Medical Center, New York, NY, USA

## Abstract

Mucormycosis is a rare and invasive fungal disease with high mortality rate caused by members of the order Mucorales. Mucorales species are vasotrophic organisms that may cause angioinvasive disease in immunosuppressed hosts. Risk factors include diabetic ketoacidosis, chronic kidney disease, organ or bone marrow transplantation, neutropenia, burns, malignancies, and steroid therapy. There are six different clinical presentations of mucormycosis, which includes rhino-orbital cerebral, pulmonary, gastrointestinal, cutaneous, disseminated, and miscellaneous infection. Here, we report a case of a 57-year-old male with stage-IV sarcoidosis on long-term steroids presenting with upper gastrointestinal bleeding and obstructive uropathy who was diagnosed with systemic mucormycosis. Biopsy obtained by endoscopy revealed necrotic debris with acute leukocytic exudate and numerous variably sized, 90-degree angulated fungal hyphae favoring mucormycosis-causing species. Imaging studies showed hydronephrosis, and cystoscopy findings were consistent with fungal infection of the bladder. Isavuconazonium sulfate was used as systemic salvage therapy along with continuous bladder irrigation with amphotericin-B for localized bladder infection after a trial with first-line systemic treatment with intravenous liposomal amphotericin-B failed. A repeat endoscopy showed inflammatory changes with a pathology report in which mucormycosis was no longer appreciated. The patient was discharged home to complete 6 months of antifungal therapy with monthly follow-ups. The patient has been asymptomatic after 12-month completion of therapy.

## 1. Introduction

Mucormycosis is an angioinvasive infectious and frequently fatal disease caused by fungi of the order Mucorales [1–3]. It is also known as zygomycosis and phycomycosis [4, 5]. The first cases of mucormycosis were reported in the early 1800s by Cohnheim and Paltauf who coined the phrase mycosis mucorina [6]. It was not until 1942 that the disease received more recognition when three cases of central nervous mucormycosis were diagnosed in diabetic patients [5]. The disease is rapidly progressive and potentially lethal and has been reported in many countries around the world [4]. These fungi are frequently found in decaying vegetative and organic matter [6, 7]. Angioinvasion and thrombosis secondary to mucormycosis cause an extensive necrosis of the affected tissue [1, 8]. Mucormycosis in one organ can spread hematogenously to other organs [3]. Infection can occur in two distinct patient settings. The first group includes patients with metabolic abnormalities such as diabetic ketoacidosis, iron overload syndromes, and chronic kidney disease; the second group includes patients with defects in innate immune cells such as neutropenia, high-dose steroid use, and organ or bone marrow transplantation [2, 4, 6]. The spectrum of mucormycosis ranges from localized rhinocerebral, sinopulmonary, and cutaneous disease to disseminated infections [3]. The most commonly isolated genera are Rhizopus spp, Mucor spp, Rhizomucor spp, and Lichtheimia spp [2, 3, 8]. Mortality associated with mucormycosis varies according to the presentation of the infection; for instance, people with sinus infections have 46% of mortality rate, 76% for pulmonary infections, and 96% for disseminated mucormycosis [3, 4, 9]. Treatment delays have been associated with an increase in the mortality of affected patients [1]. Interestingly, diagnosis of gastrointestinal mucormycosis is rare, and delay in diagnosis increases mortality rate as high as 85% [3]. Cases associating mucormycosis with patients diagnosed with sarcoidosis are even rarer. To our knowledge, there have only been few cases published thus far.

We present a case report of a 57-year-old male with history of coronary artery disease, systolic heart failure, alcohol abuse, and stage-IV sarcoidosis on long-term steroids presenting with upper gastrointestinal bleeding and obstructive uropathy diagnosed with systemic mucormycosis.

## 2. Case Presentation

**Figure 1:**
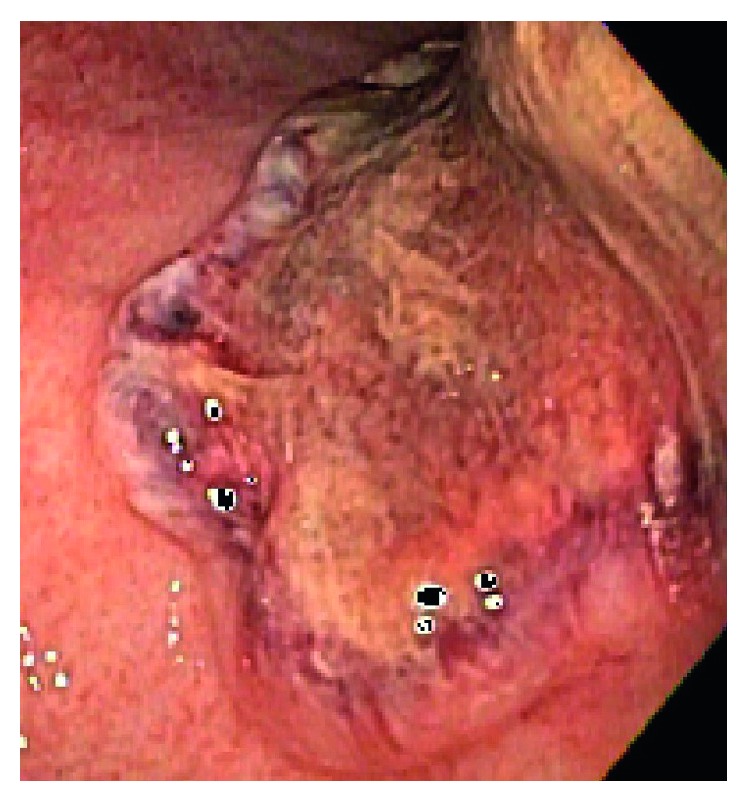
Large ulcerated lesion with no active bleeding and everted edges suspicious for malignancy.

**Figure 2:**
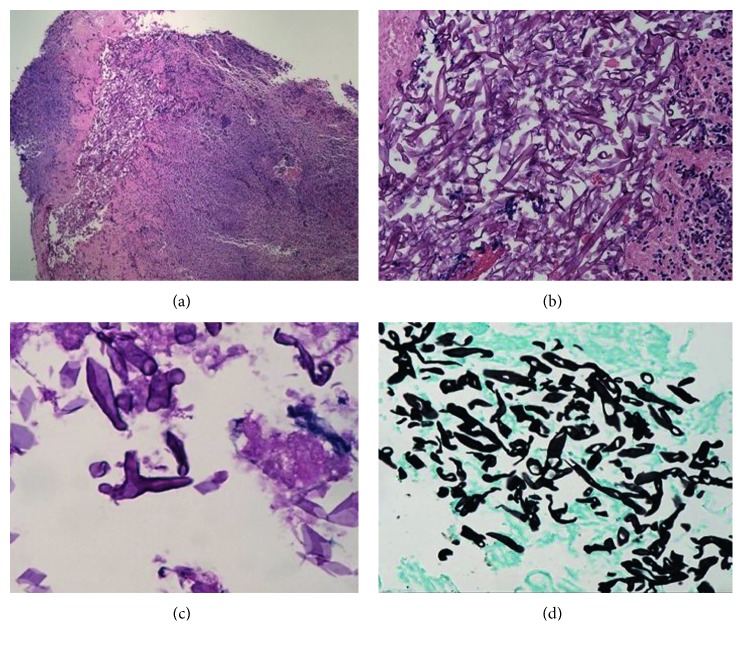
Using Gomori's methenamine silver stain, pathology report of biopsy taken from ulcerated lesion on gastric mucosa shows 90-degree angulated fungal hyphae characteristic of mucormycosis.

**Figure 3:**
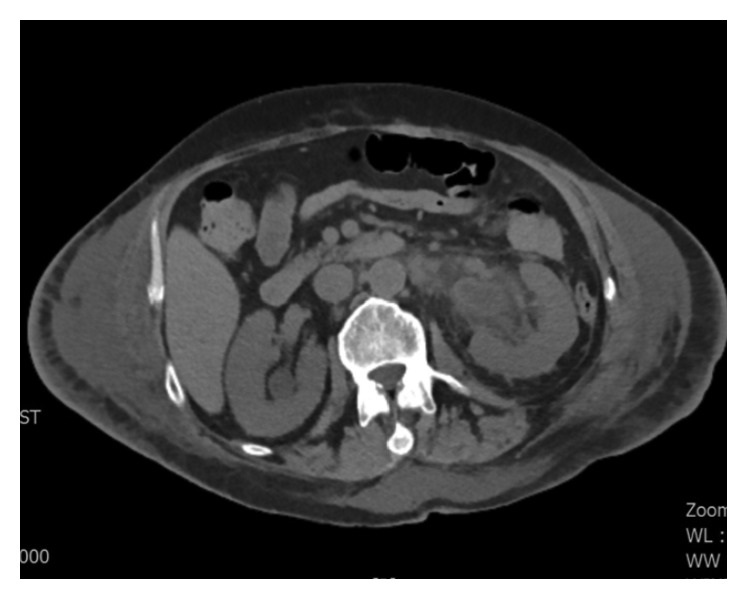
CT abdomen (transverse view) shows bilateral hydronephrosis.

**Figure 4:**
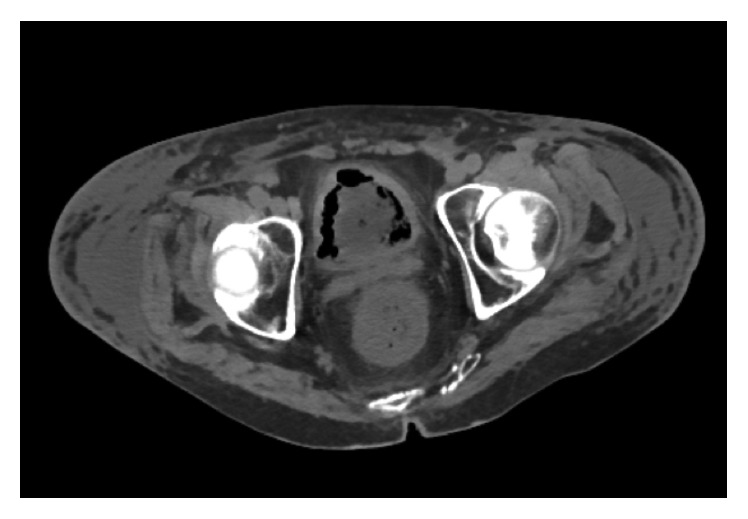
CT abdomen (transverse view) demonstrates nonspecific density and foci of air noted in bladder.

A 57-year-old African American male with past medical history of coronary artery disease, systolic heart failure, alcohol abuse, and stage-IV sarcoidosis on long-term oral prednisone at 10 mg daily for over two years presented with massive hematemesis and melena that started the night before his arrival to the Emergency Department. Upon physical examination, the patient appeared pale and was noted to have tachycardia, hypotension, and hematochezia. He denied any recent alcohol intake, chest pain, abdominal pain, and abdominal or rectal trauma. Vital signs consisted of a blood pressure of 84/33 mmHg, heart rate of 118 bpm, room air saturation of 92%, and temperature of 98.8 degrees Fahrenheit (37.1 degrees Celsius). He was admitted to the medical ICU for hypovolemic shock secondary to massive bleeding that required immediate endotracheal intubation for airway protection. An initial blood work showed a hemoglobin count of 13.9 g/dL, white blood cell count of 18.7 K/UL, platelet count of 159 K/UL, sodium of 140 mmol/L, potassium of 3.8 mmol/L, chloride of 101 mmol/L, bicarbonate below 10 mmol/L, calcium of 2.2 mmol/L, albumin of 2.7 g/dL, CRP of 233 mg/L, ESR of 36 mm/hr, BUN of 17 mg/dL (6.07 mmol/L), creatinine of 1.5 mg/dL (132.6 Umol/L), lactic acid of 17.0 mmol/L, BNP of 1140.63 pg/ml, total bilirubin of 0.4 mg/dL, troponins of 0.020 ng/mL, and CK-MB of 408 ng/mL. Resuscitation was initiated with administration of isotonic solutions, blood transfusions, and initiation of vasoactive medications, and pantoprazole drip was provided. An initial urgent endoscopy revealed a bleeding Dieulafoy lesion, which was immediately clipped. Acute kidney injury was addressed with aggressive fluid resuscitation as evident by increase in BUN of 33 mg/dL (11.78 mmol/L) and creatinine of 2.9 mg/dL (256.33 Umol/L). CT scan of abdomen performed to rule out ischemic colitis in the setting of progressive abdominal distention showed a diffuse pericolonic inflammation most pronounced at the rectosigmoid colon. Repeat endoscopy showed multiple ulcerated lesions of the gastric mucosa (Figure 1) with a biopsy that revealed necrotic debris with acute leukocytic exudate and numerous variably sized, 90-degree angulated fungal hyphae favoring mucormycosis (Figure 2). Intravenous liposomal amphotericin-B (5 mg/kg/day at 60 kg weight) was started at a dose of 300 mg daily. Due to deterioration of renal function on the second day, treatment was switched to oral suspension of posaconazole at a dose of 400 mg twice a day for a total of 6 days (of note, no levels of posaconazole were drawn during hospital course). Repeat CT scan of the abdomen showed mild-to-moderate hydroureter with hydronephrosis and bladder wall thickening Figures 3 and 4. Cystoscopy showed distorted anatomy of bladder lumen with multiple mounds of tissues all throughout the bladder most notable in the trigone with failure to locate ureteral orifices. Random biopsies taken from the bladder tissue also showed necrotic debris with active inflammatory and numerous variably sized angulated fungal hyphae consistent with Mucorales species. Once bladder biopsy confirmed Mucor spp, treatment was switched from posaconazole to intravenous isavuconazonium sulfate at a dose of 372 mg every eight hours for 48 hours as loading dose and then to an oral dose of 372 mg daily. Unfortunately, no sensitivity of the isolate to the newer triazoles was available. Surgical approach was considered; however, given patient's frail state and multiple comorbidities, such approach was deferred. Hydronephrosis significantly improved after placement of bilateral nephrostomy tubes. In addition, continuous bladder irrigation with amphotericin-B was given for localized bladder infection for a total of 5 days. A three-way Foley catheter was inserted for continuous amphotericin-B bladder irrigation where 200 ml of medication was administered via catheter; then, the catheter was clamped for 90 minutes and drained to gravity every six hours. After three weeks of systemic treatment with antifungals, a third endoscopy showed only inflammatory changes with a pathologic report, in which mucormycosis was no longer appreciated. His kidney function improved and remained stable after clamping and removal of his nephrostomy tubes. The patient completed 6 months of therapy with 372 mg of oral isavuconazonium sulfate per day. He is currently at home with resolution of his symptoms and no clinical evidence of relapse after 12 months since completion of his treatment.

## 3. Discussion

Mucormycosis infection occurs through inhalation of sporangiospores of the fungi and by direct inoculation into the skin [2, 10] such as traumatic inoculation, intravenous cannulation, and bladder catheterization [5]. Different clinical presentations may occur in susceptible hosts, including rhino-orbital cerebral, pulmonary, gastrointestinal, cutaneous, disseminated, and miscellaneous infection [2, 4, 8]. These fungi can cause thrombosis of the small and large arteries with infarction and necrosis of the affected organs [5, 7]. Disseminated mucormycosis has been reported in patients with cerebral, cutaneous, and pulmonary manifestations in 48%, 39%, and 20% of the cases, respectively [4]. Gastrointestinal mucormycosis is rare, especially in industrialized nations. However, there has been a substantial increase in the number of cases of gastric and gastrointestinal mucormycosis being diagnosed, based on the increased number of cases published between 1959 and 2011 [11]. Only 25% of cases of gastrointestinal mucormycosis are diagnosed antemortem. Some authors have described gastrointestinal presentation mainly in premature neonates; malnourished individual; and individuals with hematological malignancies, diabetes mellitus, or a history of corticosteroid use [3]. The stomach is the most commonly affected site followed by the colon and ileum [1].

The predominance of the clinical manifestations varies from host to host and by the organ affected [4]. Diagnosis requires the use of clinical, mycological, pathological, and imaging studies [2]. Standard methods used to diagnose mucormycosis include direct microscopic examination, cultures, and histopathology [8]. Definitive diagnosis is obtained through positive cultures and histopathological demonstration of the hyphae [2, 10]. Morphologic characteristics of the hyphae include a thick and nonseptate hypha branching at right-angle [2, 7]. The reported prevalence of positive cultures ranges from 50 to 75%, with a combined prevalence of both histopathological examination and positive cultures found in upto 43% of the cases [2].

Early diagnosis, correction of predisposing factors, aggressive management with surgical debridement, and appropriate antifungal therapy play a fundamental role in achieving optimal outcomes [2, 10, 12]. Current available therapies against Mucorales include amphotericin-B, posaconazole, and recent approval of isavuconazole [12]. Polyenes such as amphotericin-B remain to be the preferred therapeutic agents in the treatment of mucormycosis [4]. Before the development of amphotericin-B in the 1950's, the disease was unvaryingly fatal [5]. Delay in the initiation of amphotericin-B for more than 6 days after diagnosis has been associated to double the mortality at 12 weeks [4]. Side effects of amphotericin include fever, nausea, vomiting hypokalemia, and nephrotoxicity [6]. Mucorales treated with amphotericin-B and adjunctive surgical debridement has a mortality rate of 50 to 100% [13], being 96% in disseminated infections, 85% in gastrointestinal infections, and 76% in pulmonary affection [14].

The lipid formulations of amphotericin-B have the advantages of having a better brain penetration, reduction of fungal burden, immunomodulatory effects, and potentially less nephrotoxic effects [4]. Posaconazole is the first azole that has been shown to be effective in cases of mucormycosis that do not respond satisfactorily to amphotericin-B [6]. Combined posaconazole and amphotericin-B therapy has not offered survival benefit over amphotericin-B therapy alone [4]. Isavuconazole, with intravenous and oral administration has demonstrated partial activity against different strains of Mucorales [8].

As part of the VITAL trial, a subgroup of 37 patients with mucormycosis in a phase 3, open label, noncomparator trial demonstrated a response rate of 31.6% for primary therapy and 36.4% for salvage therapy [12]. The use of amphotericin-B bladder irrigation (ABBI) is controversial. There are limited data on the use of ABBI for treatment of mucormycosis. Therefore, the data available in the use of ABBI in the setting of candiduria were used to support its use in our patient. In patients with candiduria, continuous ABBI has demonstrated to be superior to intermittent irrigation [15]. Furthermore, the use of ABBI for more than 5 days exhibited better results than treating for less than 5 days (88% versus 78%) [16]. Other agents formerly used in the treatment of mucormycosis are iron-chelating agents such as deferasirox [1, 8]; However, its use was not longer recommended since its failure to demonstrate its safety and efficacy in the trial DEFEAT MUCOR published in 2011 [17]. Surgical debridement has shown to decrease the mortality by 79% when compared to the cases being treated with antifungal therapy only [8].

Survival after infection has been improved by techniques that optimize predisposing comorbidities, as well as by the initiation of coadjuvant therapies with immunomodulatory treatments that include the use of statins, granulocyte transfusion, and cytokines in order to set the balance between successful eradication of the invading pathogen and limitation of the damage to the affected tissue [4]. For instance, steroid therapy increases susceptibility for infections because of its effect on cellular immunity by causing a redistribution of lymphocytes from the circulation. In long-term steroid users, an indicator for severe infection could be analyzed by a decline in CD4 T count [18]. A few published literatures suggest sarcoidosis alone to be a possible risk factor for mucormycosis [19, 20]; however, more evidence is needed to support it. The length of the treatment should be individualized and continued until clinical resolution of signs and symptoms are noted. In addition, patients should have negative follow-up cultures and biopsies as well as the reversal of the underlying immunosuppressive disease if possible [1]. The overall long-term prognosis of patients who have suffered a mucormycosis infection is poor [1]. Although it is advised to monitor for clinical relapse of the disease, there are no specific validated approaches or biomarkers to achieve this goal. Patients who had suffered from this infection should be encouraged to be compliant with their medical regiments and to adopt healthy life practices. It is important to remember that surgical debridement and antifungal therapy remain to be the cornerstone of treatment.

## 4. Conclusion

Early diagnosis of mucormycosis is challenging given the diverse clinical manifestations that can indicate the presence of this infection. Our patient had multiple comorbidities that may have contributed to the uniqueness of his clinical presentation, and the individualized therapy he received helped increase his chances of survival. The correction of the underlying conditions and interventions such as surgical debridement with appropriate antifungal therapy has shown to improve the survival outcomes of affected individuals. High clinical suspicion and multidisciplinary team approaches are often required to overcome this fatal yet treatable disease.
